# Toll-like receptor 4 single-nucleotide polymorphisms Asp299Gly and Thr399Ile in head and neck squamous cell carcinomas

**DOI:** 10.1186/1479-5876-9-139

**Published:** 2011-08-21

**Authors:** Christoph Bergmann, Hagen S Bachmann, Agnes Bankfalvi, Ramin Lotfi, Carolin Pütter, Clarissa A Wild, Patrick J Schuler, Jens Greve, Thomas K Hoffmann, Stephan Lang, André Scherag, Götz F Lehnerdt

**Affiliations:** 1 Department of Otorhinolaryngology, University of Duisburg - Essen, Hufelandstrasse 55, 45127 Essen, Germany; 2 Department of Pharmacogenetics, University of Duisburg - Essen, Hufelandstrasse 55, 45127 Essen, Germany; 3 Department of Pathology, University of Duisburg - Essen, Hufelandstrasse 55, 45127 Essen, Germany; 4 Institute for Transfusion Medicine, University of Ulm, Helmholtzstr. 10, 89081 Ulm, Germany; 5 Institute for Medical Informatics, Biometry and Epidemiology, University of Duisburg - Essen, Hufelandstrasse 55, 45122 Essen, Germany

**Keywords:** Toll-like receptor 4, Single-nucleotide polymorphism, HNSCC

## Abstract

**Background:**

Chronic inflammation plays an important role in head and neck squamous cell carcinomas (HNSCC). This study addresses the impact of two single nucleotide polymorphisms (SNP) Asp299Gly and Thr399Ile of the toll-like receptor (TLR) 4 gene on the clinical outcome while accounting for the influence of adjuvant systemic therapy in a large cohort of HNSCC patients.

**Methods:**

Genotype analysis was done using DNA from tissue samples from 188 patients with HNSCC; TLR4 protein expression was assessed immunohistochemically in tissue microarrays. Classical survival models were used for statistical analyses.

**Results:**

Ten percent of patients with HNSCC presented with the *TLR4 *299Gly and 17% with the *TLR4 *399Ile allele. Patients with the heterozygous genotype *TLR4 *Asp299Gly had a significantly reduced disease-free and overall survival. Also, patients with the heterozygous genotype *TLR4 *Thr399Ile had a reduced disease-free survival. Notably, these associations seem to be attributable to relatively poor therapy response as e.g. reflected in a significantly shorter DFS among HNSCC patients carrying the Asp299Gly variant and receiving adjuvant systemic therapy.

**Conclusion:**

According to this study, TLR4 299Gly und 399Ile alleles may serve as markers for prognosis of head and neck cancer in patients with adjuvant systemic therapy, particularly chemotherapy, and might indicate therapy resistance.

## Background

The functional relationship between inflammation and cancer has been described since 1863, at first by Virchow [1]. Many cancers arise from sites of chronic inflammation, where inflammatory cells orchestrate the tumor microenvironment fostering neoplastic processes like proliferation, survival, and migration [2]. The upper aero-digestive tract is chronically exposed to pathogens and toxic irritants. For example, human papilloma virus 16 DNA can be detected in up to 72% of oropharyngeal cancers [3]. Further, tobacco and alcohol consumption is implicated in 75% of head and neck squamous cell carcinomas (HNSCC) [4,5]. Thus, infection and inflammation critically impact the development of HNSCC [6].

The family of mammalian Toll-like receptors (TLR) consists of 11 members and is mainly expressed on innate immune cells [7]. TLR play a pivotal role in immune responses to exogenous pathogen-associated (PAMPs) or to endogenous danger-/damage-associated molecular patterns (DAMPs). However, TLR are also expressed on endothelial and epithelial cells, including tumor cells [8,9]. To date, little is known about the function and the biological importance of TLR expressed on tumor cells. Preliminary evidence suggests that TLR expressed on tumor cells may play an important role in the tumor development. It has been proposed that TLR-signaling mediated infection- or injury-induced inflammation can promote tumorigenesis owing to chronic tissue damage with subsequent induction of deregulated tissue repair [10].

TLR4 is a well characterized TLR family member, which recognizes PAMPs (e.g. lipopolysaccharide - LPS, a component of gram-negative bacterial wall component) and DAMPs (e.g. high-mobility group box 1 - HMGB1, a highly conserved ubiquitous protein with pro-inflammatory cytokine-like properties) [11]. TLR4 expression has also been described on tumor cells of HNSCC, where its level of expression correlates with tumor grade. Further, TLR4 ligation on HNSCC cells with LPS induced tumor promotion by enhancing proliferation, activation of NFκB and resistance to NK cell mediated cytotoxicity [12].

In 2001, Arbour *et al. *identified germ-line single-nucleotide polymorphisms (SNPs) with co-segregating missense mutations. These SNPs are an A/G transition in exon3 causing an aspartic acid/glycine substitution at amino acid location Asp299Gly (rs4986790), and a C/T transition in exon4 of *TLR4 *causing a threonine/isoleucine switch at amino acid location Thr399Ile (rs4986791). These polymorphisms alter the amino acid sequence of the TLR4 protein and affect the extracellular domain and ligand-recognition area of the TLR4 receptor. These SNPs have been reported to be associated with a blunted response to inhaled LPS in humans [13]. Importantly, Apetoh et al. reported that patients with breast cancer, who carry at least one TLR4 loss-of-function allele, relapse more quickly after radiotherapy and chemotherapy than those carrying two wildtype TLR4 alleles. They also demonstrated that TLR4 Asp299Gly SNP reduces the interaction between TLR4 and the endogenous danger signal HMGB1. The latter resulted in reduced capacity of dendritic cells to cross-present melanoma cells to Mart1-specific cytotoxic T cells [14]. Also, both TLR4 polymorphisms are linked with an increased susceptibility for gastric cancer and gallbladder cancer [15,16]. In aggregate, these results delineate a clinically relevant pathway triggered by tumor cells with an altered TLR4 SNP.

Here, we investigate the relevance of *TLR4 *SNPs Asp299Gly and Thr399Ile in 188 HNSCC patients prospectively with a long follow-up (50 months) and complete representative adjuvant therapy (chemotherapy and radiation). In addition, TLR4 expression is analyzed by immunohistochemistry (IHC) next to *TLR4 *SNP genotype in HNSCC patients. Moreover, we investigated the influence of adjuvant systemic therapy on prognostic impact of TLR4.

## Methods

### Patients and Tissue Samples

Tissue specimens of 188 consecutive HNSCC were collected by the Department of Pathology, University hospital Essen, Germany. All patients were diagnosed and treated at the Department of Otorhinolaryngology, University Hospital Essen, Germany (1995-2002); treatment decisions were based on consensus recommendations from oncologists, radiotherapists and head and neck surgeons, which were based on treatment guidelines of treatment at the time. All patients gave written informed consent for research use of the tissues and for participating in the research project. The study was conducted according to the Declaration of Helsinki. Tissues were obtained during diagnostic or therapeutic surgery.

Overall, ninety nine (53%) patients received cisplatin/5-fluorouracil-based chemotherapy regimens and radiation up to 70 Gy as adjuvant therapy after surgery. Seventeen (9%) patients received primary radio-chemotherapy. Follow-up was performed regularly; median follow-up in patients still alive at analysis was 50 months (range, 0 to 128 months). Relapse data were available for all patients: 60 (32%) experienced disease recurrence and 89 (47%) death. Complete therapeutic regimens are listed in Table 1 and 2.

**Table 1:** Associations between TLR4 Asp299Gly SNP genotype and clinicopathological variables

	Total	Asp299Asp	Asp299Gly	*P*
n (%)	138	125 (90.6)	13 (9.4)	
Oro-Hypopharyngeal SCC; n (%)	37	34 (91.9)	3 (8.1)	0.76
Laryngeal SCC; n (%)	101	90 (89.1)	11 (10.9)	
Mean age ± SD [years]	61 ± 10	60 ± 10	63 ± 13	0.66
Median follow up [months] (range)^#^	50 (0-129)	52 (0-129)	42 (8-98)	0.37
Sex (male/female); n	119/19	106/19	13/0	0.21
Smoking; n (%)	124 (89.8)	112 (89.6)	12 (92.3)	1.00
Mean pack years ± SD	45 ± 25	45 ± 24.6	50 ± 29.6	0.62
Primary therapy				0.02
Surgery alone; n (%)	61	57 (45.6)	4 (30.8)	
Surgery + RCT^§^; n (%)	54	51 (40.8)	3 (23.1)	
Primary RCT^§^; n (%)	23	17 (13.6)	6 (46.1)	
AJCC stage				0.53
I; n (%)	25	22 (17.6)	3 (23.1)	
II; n (%)	33	30 (24.0)	3 (23.1)	
III; n (%)	25	22 (17.6)	3 (23.1)	
IVA; n (%)	50	47 (37.6)	3 (23.1)	
IVB; n (%)	3	2 (1.6)	1 (7.6)	
IVC; n (%)	2	2 (1.6)	0 (0.0)	
Grade				0.32
1; n (%)	9	7 (5.6)	2 (15.4)	
2; n (%)	96	87 (69.6)	9 (69.2)	
3-4; n (%)	25	23 (18.4)	2 (15.4)	

^# ^as based on the observed data (ignoring censoring); ^§^RCT: radiation + chemotherapy

**Table 2:** Associations between *TLR4 *Thr399Ile SNP genotype and clinicopathological variables

	Total	Thr399Thr	Thr399Ile	*P*
n (%)	62	51 (82.3)	11 (17.7)	
Laryngeal SCC; n (%)	62	51 (82.3)	11 (17.7)	
Mean age ± SD [years]	60 ± 10	61 ± 10	57 ± 7	0.13
Median follow up [months] (range)^#^	52 (0-129)	55 (0-129)	43 (9-98)	0.38
Sex (male/female); n	55/7	44/7	11/0	0.33
Smoking; n (%)	54 (87.1)	43 (84.3)	11 (100)	0.33
Mean pack years ± SD	50 ± 20.3	48.9 ± 20.3	54.1 ± 21.1	0.53
Primary therapy				0.02
Surgery alone; n (%)	34	31 (60.8)	3 (27.3)	
Surgery + RCT^§^; n (%)	23	18 (35.3)	5 (45.4)	
Primary RCT^§^; n (%)	5	2 (3.9)	3 (27.3)	
AJCC stage				< 0.01
I; n (%)	11	10 (19.6)	1 (9.1)	
II; n (%)	16	14 (27.5)	2 (18.2)	
III; n (%)	9	3 (5.9)	6 (54.5)	
IVA; n (%)	25	23 (45.1)	2 (18.2)	
IVB; n (%)	0	0 (0.0)	0 (0.0)	
IVC; n (%)	1	1 (1.9)	0 (0.0)	
Grade				0.86
1; n(%)	4	4 (7.8)	0 (0.0)	
2; n(%)	43	34 (66.6)	9 (81.8)	
3-4; n(%)	11	9 (17.6)	2 (18.2)	

^# ^as based on the observed data (ignoring censoring); ^§^RCT: radiation + chemotherapy

Due to poor or lack of sufficient material for PCR or IHC or absence of complete clinicopathological data, the initial sample of 188 patients of the total collective was split into three groups: a group of 138 for analysis of *TLR4 *Asp299, a group of 62 for analysis of *TLR4 *Thr399 (39 patients were analyzed for both SNPs), and a group of 78 patients with HNSCC for TLR4 expression analysis (43/78 were also genotyped for *TLR4 *Asp299; 20/78 for *TLR4 *Thr399 - see Table 3).

**Table 3:** Comparison of *TLR4 *genotype and *TLR4 *expression

SNP	*TLR4 *expression	Total	wild-type genotype (Asp299Asp or Thr399Thr)	heterozygous genotype (Asp299Gly or Thr399Ile)	*P*
*TLR4 *Asp299Gly (rs4986790)	0	11	10	1	0.42
	1	7	7	0	
	2	21	16	5	
	3	4	3	1	
					
*TLR4 *Thr399Ile (rs4986791)	0	1	1	0	1.00
	1	1	1	0	
	2	15	12	3	
	3	3	3	0	

### Immunohistochemistry

Routinely formalin-fixed and paraffin-embedded tumor tissue blocks were retrieved from the files of the Institute of Pathology (University Hospital of Essen, Germany) and processed using the tissue microarray (TMA) technology. In short, tumor tissue cores of 3 mm in diameter were removed from the area of interest from each donor block using a hollow needle skin biopsy punch (PFM, Cologne, Germany) and inserted into recipient blocks in a precisely spaced, array pattern. One tissue core of each normal thyroid and kidney tissues in preset position in each block served as control tissue and helped with the orientation.

5 μm TMA sections were cut and mounted on SuperFrost^® ^Plus slides (Menzel, Braunschweig, Germany). IHC was performed using the Dako Autostainer Plus System (DakoCytomation, Carpinteria, CA, USA). After antigen retrieval (water bath at 95°C; 20 min in citrate buffer), TMA slides were immunostained by the TLR4 (H-80) rabbit polyclonal antibody (sc-10741, dilution 1:100, Santa Cruz Biotechnology Inc., Sant Cruz, CA, USA). Antibody visualisation was performed using the anti-mouse IgG detection kit (EnVision+, DakoCytomation, Carpinteria, CA, USA) according to the manufacturer's recommendations.

### Evaluation of immunohistochemical staining

**Figure 1:**
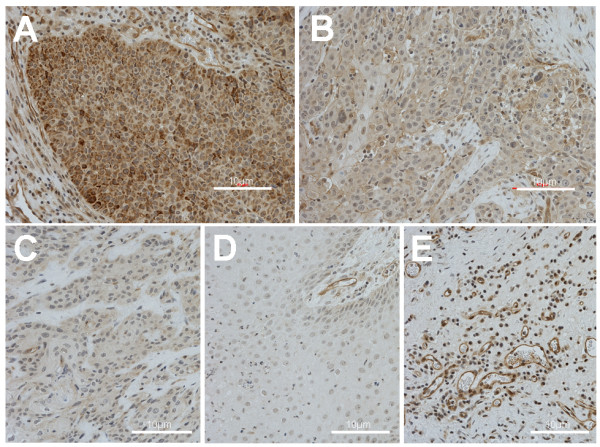
***TLR4 *immunohistochemistry in head and neck squamous cell carcinomas**. (A) Strong (score 3+); (B) moderate (score 2+); (C) weak staining (score 1+); (D) negative control (no immunoreactivity); (E) positive control (strong staining in endothelial inflammatory cells expressing TLR4).

Stained sections were reviewed by one of the authors (AB). The percentage of tumor cells showing a positive membranous/cytoplasmatic staining and the intensity of staining were assessed. Cases with complete lack of staining were scored as negative, a weak membranous/cytoplasmic reaction in 1-50% was classified as 1+, moderately strong reactions in up to 80% of tumor cells were scored 2+, whereas moderate to strong membranous/cytoplasmic immunostaining of > 80% of tumor cells were classified as 3+ (Figure 1). Inherent positivity of capillary endothelial cells and mononuclear inflammatory cells in the stroma served as positive control; for negative control purposes the incubation step with the primary antibody was omitted.

### Sequence analysis of TLR4

As described earlier [17], DNA samples were extracted from 10- μm sections of formalin-fixed, paraffin-embedded tumor tissue. The germline mutations *TLR4 *Asp299Gly (rs4986790) and Thr399Ile (rs4986791) were analyzed in all patients using polymerase chain reaction restriction fragment length polymorphism (PCR-RFLP). For rs4986790 (*TLR4 *8552A > G), PCR was performed with the forward primer 5'-CTG CTC TAG AGG GCC TGT G-3' and the reverse primer 5'-TTC AAT AGT CAC ACT CAC CAG-3', resulting in a 140 bp fragment. After denaturation at 95°C, 38 cycles of DNA amplification were performed using Taq DNA Polymerase 2× Master Mix RED (Ampliqon-Biomol, Hamburg, Germany) at 95°C for 30 s, 61°C for 30 s and 72°C for 30 s. Digestion with BccI at 37°C (New England Biolabs Inc., Ipswich, MA, USA) and results in fragments of 77 bp and 63 bp for the G-allele vs. 140 bp for the A-allele (no digestion) separated on a 2.5% agarose gel were analysed. To genotype for rs4986791 (*TLR4 *8852C > T), PCR was performed with the forward primer 5'-CTA CCA AGC CTT GAG TTT CTA G-3' and the reverse primer 5'-AAG CTC AGA TCT AAA TAC CT-3'. After denaturation at 95°C, 38 cycles of DNA amplification were performed using Taq DNA Polymerase 2× Master Mix RED (Ampliqon-Biomol, Hamburg, Germany) at 95°C for 30 s, 53°C for 30 s, and 72°C for 30 s. The resulting 110 bp PCR products were digested using the restriction enzyme BslI at 55°C and analyzed on a 2.5% agarose gel. The unrestricted products represent the TT genotype; the completely restricted products (89 and 21 bp) represent the CC genotype.

Electrophoresis was performed using SYBR Safe^® ^DNA Gel Stain (Invitrogen Corporation, Carlsbad, CA, USA) for visualization under UV light. Correctness of genotyping has been ensured by concomitantly analyzing DNA samples from human volunteers whose genotypes have already been confirmed by direct sequencing. Re-genotyping of both polymorphisms in 40 randomly chosen samples revealed complete concordance with previous results.

While the *TLR4 *Asp299Gly genotype was evaluable in 138 patients, the *TLR4 *Thr399Ile genotype was only evaluable in 62 patients. This was due to a low amount of and strongly degraded DNA in the available paraffin-embedded tumor tissue probably because of unbuffered paraffin on the tumor cells in more than 10 years old paraffin-embedded tissue samples or a high guanine-cytosine content in the gene region for Thr399, which hampers amplification. Therefore every sample was tested four times but utilizable DNA-products were available only for those 62 patients. Due to the reduced quality of samples other methods for genotyping (e.g. direct sequencing, pyrosequencing or TaqMan-genotyping) were not considered.

### Statistical Analysis

The two genotype distributions were tested for deviations from Hardy Weinberg equilibrium (both two-sided exact p-values were 1.0). Associations between clinical tumor characteristics and *TLR4 *genotype were assessed either by non-parametric Wilcoxon-Mann-Whitney tests in case of quantitative variables or by generalized Fisher's exact test for categorical variables in 2 × m tables. Time to events was calculated as the difference between primary diagnosis and either the date of the clinical assessment where the respective event occurred or last clinical assessment in case of censoring. While survival probabilities were graphically assessed by the Kaplan Meier method (including a log-rank test for inference in the figures), uni- and multiple cox regression analyses were used for the statistical analyses. In the multiple regression model variables with p > .1 in the univariate model were excluded to address estimation concerns. Model diagnostic of the proportional hazards (PH) assumption for the *TLR4 *genotypes comprised both graphical and formal investigations - none of which indicated strong evidence for a deviation from the PH assumption. Confidence intervals were calculated with coverage of 95% level (95%CI) and accordingly the level α for each test was 0.05 (two-sided). Unless otherwise mentioned, all reported p-values are nominal and two-sided.

## Results

### Distribution of TLR4 Asp299Gly and Thr399Ile

In the present primary HNSCC cohort, 125 patients (90.6%) showed a homozygous *TLR4 *genotype for aspartate at aminoacid location 299, and 13 patients (9.4%) had a *TLR4 *Asp299Gly variant (minor allele frequency (MAF) ~4.7%). We observed no evidence for a deviation from Hardy-Weinberg equilibrium (HWE; p = 1.0; two-sided exact test). The genotype distribution is in accordance with previous reports [13,15], which describe a carrier frequency of ~7% in both healthy controls and gastric cancer patients of the Caucasian population. Regarding the other SNP (Thr399Ile) 51 out of 62 (82.3%) of our patients were homozygous for threonine and 11 heterozygous (17.7%) for threonine and isoleucine alleles (MAF ~ 8.9%; p = 1.0; two-sided exact test for deviations from HWE).

No evidence for associations was found between clinical tumor characteristics or histopathological characteristics and *TLR4 *Asp299Gly genotype (Table 1). For the *TLR4 *Thr399Thr genotype the explorative statistical analysis indicated a positive correlation between AJCC tumor stage and Thr399Thr genotype only (p < 0.01; Table 2).

### Expression patterns of TLR4

Sixteen percent of HNSCC tumors showed low (score 1+), 49% moderate (2+), 9% strong (3+), and 26% showed no TLR4 staining (Figure 1; Table 3). TLR4 staining (all scores) showed a diffuse and fine granular cytoplasmatic pattern. Distinct membrane staining was observed in some tumors but never without cytoplasmatic staining. TLR4 scores did not significantly correlate with clinicopathologic variables, in particular there was no correlation between TLR4 expression patterns and disease-free or overall survival (data not shown).

### TLR4 Genotype and Expression of TLR4

*TLR4 *genotype showed no evidence for an association with TLR4 protein expression phenotype (IHC; Table 3). Altered grouping of the expression values (low/high for grade 0/1 or 2/3) or *TLR4 *genotype (wild-type for both SNPs vs. any heterozygous variant) had no impact on this observation.

### TLR4 Genotype and Disease Advancement

**Figure 2:**
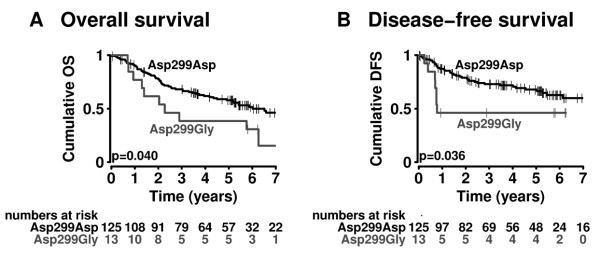
***TLR4 *Arg299 allele impact on survival and tumor recurrence**. Probability of (A) overall survival (OS) and (B) disease-free survival (DFS) in patients according to *TLR4 *allele status (*TLR4 *Asp299Asp vs. *TLR4 *Asp299Gly). P-values obtained from the log-rank test are indicated.

Our analysis revealed a significant association between *TLR4 *Asp299Gly genotype and recurrence of disease with a hazard ratio (hr) of 2.37 for a reduced disease-free survival (DFS; 95%CI: 1.05-5.33; p = 0.04; Figure 2A). Also, overall survival (OS) was significantly associated with Asp299Gly genotype with a hazard ratio of 2.00 for reduced survival (OS; 95%CI: 1.02-3.92; p = 0.04; Figure 2B; Table 4).

**Table 4:** Uni- and multivariate cox model for overall survival including clinicopathological variables and *TLR4 *Asp299Gly SNP genotype - hazard ratio point estimates, 95% CIs and p-values (2-sided) from Wald-tests are reported

	Univariate cox model		Multivariate cox model*	
		
	hazard ratio [95% CI]	*P*	hazard ratio [95% CI]	*P*
*TLR4 *Asp299Gly genotype				
Asp299Asp	1	-	1	-
Asp299Gly	2.00 [1.02...3.92]	0.04	2.02 [1.01...4.06]	0.05
Age				
[per 5 years]	1.11 [0.98...1.25]	0.10		
Sex				
female	1	-	1	-
male	2.55 [1.03...6.36]	0.04	2.91 [1.15...7.32]	0.02
Smoking^#^				
no	1	-		
yes	0.91 [0.42...2.00]	0.82		
AJCC stage				
I	1	-	1	-
II	1.86 [0.70...4.97]	0.21	1.87 [0.70...5.00]	0.21
III	2.40 [0.89...6.50]	0.08	2.25 [0.83...6.11]	0.11
IV^§^	4.08 [1.72...9.66]	1.1 × 10^-3^	4.66 [1.96...11.09]	5.0 × 10^-4^

^# ^using 'Mean pack years' instead had no impact on the findings; ^§ ^which summarizes stages IVA, IVB and IVC

**Figure 3:**
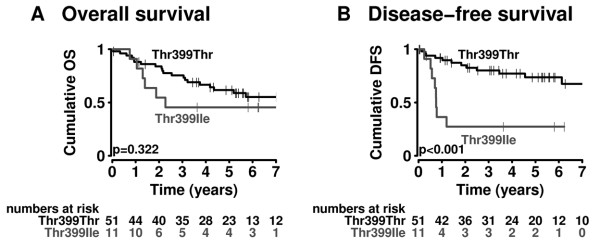
***TLR4 *Thr399 allele impact on survival and tumor recurrence**. Probability of (A) overall survival (OS) and (B) disease-free survival (DFS) in patients according to *TLR4 *allele status (*TLR4 *Thr399Thr vs. *TLR4 *Thr399Ileu). P-values from the log-rank test are indicated.

For the other SNP a similar pattern was observable (Figure 3); in case of DFS patients with the Thr399Ile variant displayed a significantly higher risk for disease advancement (hr = 4.97; 95%CI: 2.00-12.37; p = 0.0006; Figure 3B).

### TLR4 Genotype in a Multivariable Cox Regression Model

Next, we considered clinicopathological variables (age, sex, smoking, AJCC stage) in univariate cox models for overall survival. Afterwards we jointly included clinicopathological variables in addition to *TLR4 *Asp299Gly genotype status in a multivariable cox model (Table 4). Though a similar result pattern was observed for the *TLR4 *Thr399Ile variant, we decided to limit the displayed analyses to *TLR4 *Asp299Gly due to the too small sample size for the Thr399Ile variant. Even after correcting for clinicopathological variables *TLR4 *Asp299Gly genotype status was an independent prognostic factor of overall survival with a hazard ratio of 2.02 for reduced survival (95%CI: 1.01-4.06; p = 0.05; Table 4).

### TLR4 Asp299 Genotype and Adjuvant Systemic Therapy

**Figure 4:**
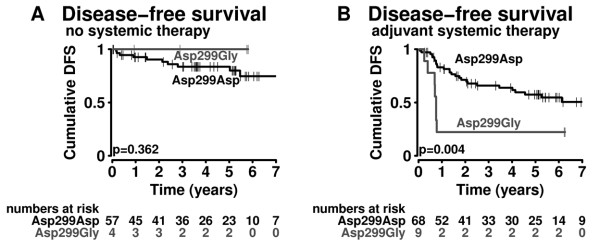
***TLR4 *Arg299 allele impact on tumor recurrence stratified by adjuvant systemic therapy**. (A) no systemic therapy and (B) adjuvant systemic therapy; in patients according to *TLR4 *allele status (*TLR4 *Asp299Asp vs. *TLR4 *Asp299Gly). P-values from the log-rank test are indicated. DFS: disease-free survival.

Based on the observed correlation of *TLR4 *genotype and applied primary therapy (Table 1 and 2), we also explored the additional impact of the use of adjuvant systemic therapy in the survival analysis (as main and interaction effect with *TLR4 *Asp299 genotype in the multivariate model of Table 4). According to this analysis, the interaction term indicated no evidence for an interaction (p = 0.18) which most likely reflects that the sample was statistically underpowered to detect an interaction. Displaying the relationship between *TLR4 *Asp299Gly genotype, use of adjuvant systemic therapy and course of disease graphically, we observed no evidence for significant survival differences between *TLR4 *genotypes in patients without adjuvant systemic therapy. However, with adjuvant systemic therapy, patients with wild-type genotype showed significantly longer DFS (p = 0.004 by log-rank test; Figure 4).

## Discussion

TLR4 signaling is strongly involved in inflammatory processes. HNSCC is a cancer entity which is known to develop from chronic inflammation [6]. Consequently, inflammation-related signaling pathways are involved the tumor and the host cells. Here, we demonstrate that TLR4 is upregulated in tumors from HNSCC patients, which is in accordance with published data [12]. The SNPs Asp299 and Thr399 have been reported to be involved in inflammation, atherogenesis, sepsis and cancer [13-15,18-21]. In this study, we provide evidence in a sample of 188 patients that these SNPs are involved in the tumor development of HNSCC with a significant impact on tumor advancement and survival of patients. Further, we demonstrate that the clinical impact of the SNP genotype is stronger if adjuvant systemic therapy is administered.

No significant associations were found between TLR4 expression status and established clinicopathological variables, in contrast to observations by Szczepanksi *et al*, who described a correlation of TLR4 expression intensity and tumor grade in a cohort of 39 HNSCC patients [12]. This group further demonstrated a TLR4-mediated protective effect for HNSCC cells from cisplatin-induced apoptosis by *in vitro *studies.

TLR4 alleles Asp299 and Thr399 may also be in linkage disequilibrium with other genetic changes that contribute to poor prognosis in HNSCC [22]. Yet, cancer cells ectopically expressing TLR4 do possess increased cell motility and invasiveness, both characteristic of an aggressive tumor phenotype [12]. We report a reduced disease-free survival and overall survival for TLR4 loss-of-function carriers in HNSCC patients. This is in line with a recently published study which gained similar results in an analysis of patients with colon cancer [23]. We show that late stage tumor progression may be genetically linked to the TLR4 Thr399Ile genotype, which is in contrast to observations of Pandey *et al*., who reported a significant association of this genotype with cervical cancer at an early stage [24].

The impact of conventional anticancer chemotherapy not only affects the tumor but also modulates the relationship between the tumor and the immune system. Recent insights are providing evidence for this new concept of cancer therapy and immunotherapy which is rapidly emerging. Chemotherapy can stimulate the immune system, either via a direct effect on immune effectors or regulatory mechanisms or indirectly, by causing lymphopenia followed by homeostatic proliferation of immune effectors that may be particularly active in the anticancer response. Interaction of TLR4 binding partners, which have been secreted by tumor cells (so-called danger signals, e.g. HMGB1) activate leukocytes through the differential engagement of multiple surface receptors like TLR4 and RAGE [25]. Further, it has been demonstrated that the TLR4 Asp299 polymorphism affects the binding of HMGB1 to TLR4 and predicts early relapse after chemotherapy in breast cancer patients. In particular, the *TLR4 *mutation has been identified as an independent predictive factor for the success of anthracycline-based adjuvant regimen'[14]. Apetoh et al. further demonstrated that HMGB1 released from oxaliplatin-treated dying tumor cells binds to TLR4 on dendritic cells and is required for cross-presentation of tumor antigens and a subsequent effective anti-tumor immune response. This effect was impaired in HeLa cells transfected with a cDNA encoding the Asp299Gly allele of TLR4 and resulted in impaired nuclear factor-κB activation after stimulation with recombinant HMGB1 [26,27].

It is also believed that optimal therapeutic effects require the immunoadjuvant effect of DAMPs like HMGB1 released from tumor cells damaged by cytotoxic anticancer agents. In other words, anticancer immune responses may contribute to the control of cancer after conventional chemotherapy. Thus, radiotherapy and some chemotherapeutic agents can induce specific immune responses that result either in immunogenic cancer cell death or in immunostimulatory side effects [28]. Very recently, Tesniere *et al. *demonstrated that Cisplatin was efficient in triggering HMGB1 release in colon cancer cells [23]. Another effect has been demonstrated for the use of anti-tumor cytotoxic agents, like oxaliplatin and 5-fluorouracil which at least partially deplete or transiently inactivate tumor-protective regulatory T cells (Treg) [29,30] as we have recently reported a significantly increased expression of TLR on Treg in patients with HNSCC [31]. Consequently, a decreased interaction of tumor-derived HMGB1 with TLR4-expressing Treg might result in a decreased anti-tumor immune response in TLR4 Asp299Gly or Thr399Ile carriers which may result in a reduced DFS and OS.

## Conclusion

Our study provides evidence for an established concept of altered chemosensitivity of tumor cells to chemotherapeutic drugs in regards to their respective polymorphic genotype [32] as we demonstrate that patients with TLR4 Asp299 wild-type genotype showed significantly better DFS with adjuvant systemic therapy including agents like cisplatin and 5-fluoruracil. Several studies have reported that SNP genotypes are highly associated with altered drug response and impact on survival (i.e. soft-tissue sarcoma [33] and colorectal cancer [34]. Ultimately, consideration of therapeutically relevant SNP might contribute to improved therapies and patients' survival. However, our study has clear limitations due to the small sample size. Therefore, clinical applicability of this biomarker information requires the inclusion of genotype information in prospectively planned randomized controlled trials (RCTs) of proper sample size in various populations.

In summary, our data suggests that polymorphisms *TLR4 *Asp299Gly and TLR4 Thr399Ile are involved in the advancement of HNSCC. Moreover, *TLR4 *genotype seems to have an impact on the success of antitumor therapy. Since TLR, and in particular TLR4, are in focus of molecular cancer therapy development [35], such results might open the door to set up prospectively planned RCTs that include *TLR4 *genotype information while evaluating new and advanced treatments of HNSCC. In the end, our observations may result in benefit for the patient when clinically exploited to enhance the efficiency and immunogenicity of current chemotherapeutic regimens as well as overcoming the immune defect induced by deficient TLR4 signaling by combining chemotherapy with alternate TLR4 agonists.

## Abbreviations

(HNSCC): Head and neck squamous cell carcinomas; (TLR): Toll-like receptors; (PAMPs): pathogen-associated molecular patterns; (DAMPs): danger-/damage-associated molecular patterns; (LPS): lipopolysaccharide; (HMGB1): high-mobility group box 1; (SNP): single-nucleotide polymorphism; (IHC): immunohistochemistry; (TMA): tissue microarray; (PCR-RFLP): polymerase chain reaction restriction fragment length polymorphism; (PH): proportional hazards; (HWE): Hardy-Weinberg equilibrium; (MAF): minor allele frequency; (AJCC): American Joint Committee of Cancer; (DFS): Disease-free survival; (OS): Overall Survival; (RAGE): receptor of advanced glycation endproducts; (RCT): Radio-Chemo-Therapy

## Competing interests

The authors declare that they have no competing interests.

## Authors' contributions

CB designed the study and participated in data analysis and interpretation. AB, TKH, SL, RL and GL provided study materials or patients. HSB, PS, JG, AB, CW and GL participated in collection and assembly of data. CP and AS participated in data analysis and interpretation. CB, HSB, AB and AS wrote the manuscript. All authors read and approved the final manuscript
